# Early systemic heparin exposure is associated with lower mortality, reduced disseminated intravascular coagulation, and fewer thrombotic complications in septic shock

**DOI:** 10.3389/fmed.2026.1931895

**Published:** 2026-09-21

**Authors:** Philipp Bernd Radermacher, Buntaro Fujita, Carl Vahldieck

**Affiliations:** 1 Department of Anesthesiology and Intensive Care Medicine, University Medical Centre Schleswig-Holstein Campus Luebeck, Luebeck, Germany; 2 Department of Cardiac and Thoracic Vascular Surgery, University Medical Centre Schleswig-Holstein Campus Luebeck, Luebeck, Germany; 3 DZHK (German Research Centre for Cardiovascular Research), Partner Site North, Luebeck, Germany; 4 Institute of Physiology, University of Luebeck, Luebeck, Germany

**Keywords:** anticoagulation, critical care, disseminated intravascular coagulation, heparin, immunothrombosis, septic shock, thrombo-inflammation, thrombosis

## Abstract

**Introduction:**

Septic shock is characterized by thrombo-inflammatory dysregulation and disseminated intravascular coagulation (DIC), contributing to organ failure and mortality. The clinical impact of heparin in septic shock remains uncertain. This study evaluated the association between early systemic heparin exposure and clinical outcomes.

**Methods:**

A retrospective multicenter cohort study was conducted using the TriNetX federated electronic health record network. Adult patients with septic shock receiving early systemic heparin exposure were compared with patients without heparin exposure. Propensity score matching balanced demographics, comorbidities, infection characteristics, organ dysfunction markers, and severity-related variables. Outcomes included mortality, DIC, thrombotic complications, renal outcomes, hematologic complications, and bleeding-related events. Clinical outcomes were assessed over a predefined 30-day follow-up period using risk-based and time-to-event analyses.

**Results:**

A total of 105,472 patients per cohort were included. Early systemic heparin exposure was associated with lower mortality (15.3% vs. 20.8%; *p* < 0.001) and lower rates of DIC (*p* < 0.001). Thrombotic complications, including ischemic stroke, acute myocardial infarction, overall thrombotic events, and arterial or venous thrombosis, occurred less frequently among heparin-exposed patients (all *p* < 0.001). In contrast, heparin exposure was associated with higher rates of acute kidney injury, renal replacement therapy, thrombocytopenia, major bleeding, and transfusion requirement (all *p* < 0.01). Kaplan–Meier analyses were consistent with risk-based analyses.

**Discussion:**

Early systemic heparin exposure was associated with lower mortality, reduced DIC, and fewer thrombotic complications, but increased rates of renal dysfunction and bleeding-related adverse events. These findings highlight a clinically relevant trade-off between potential benefit and harm. Prospective randomized studies are needed to define optimal patient selection and treatment timing.

## 1 Introduction

Sepsis and septic shock remain major causes of intensive care morbidity and mortality despite improvements in early recognition and organ support strategies. Current guidelines emphasize source control, antibiotics, and hemodynamic resuscitation, but do not recommend routine disease-modifying anticoagulation because evidence remains inconsistent (1).

Endothelial dysfunction and thrombo-inflammatory dysregulation are central features of sepsis and critical illness. Endothelial injury, including disruption of the endothelial surface layer and glycocalyx, promotes capillary leak, leukocyte adhesion, platelet activation, dysregulation of local anticoagulant pathways, microvascular thrombosis, tissue hypoperfusion, and organ dysfunction (2–6).

Septic shock is characterized by particularly severe microcirculatory failure. Immunothrombosis, disseminated intravascular coagulation (DIC), platelet activation, endothelial injury, and microvascular thrombosis contribute to impaired tissue perfusion, organ dysfunction, and death.

These abnormalities make septic shock a clinically relevant setting in which therapies targeting coagulation-related pathways may have effects beyond conventional anticoagulation (5, 7–10).

Beyond microvascular thrombosis, sepsis is associated with clinically overt cardiovascular and thrombotic events, including acute myocardial infarction (AMI), ischemic stroke, and venous thromboembolism, reflecting the systemic prothrombotic milieu generated by inflammation, endothelial dysfunction, and dysregulated coagulation (11, 12).

Heparin and related glycosaminoglycans may modulate thrombo-inflammatory pathways, attenuate leukocyte-platelet interactions, and influence processes involved in microvascular thrombosis and coagulation dysregulation (13, 14). These observations provide a biologically plausible rationale for evaluating whether systemic heparin exposure is associated with clinically relevant cardiovascular outcomes in septic shock.

Clinical evidence on heparin in sepsis remains heterogeneous. Observational studies and meta-analyses report signals of reduced mortality and DIC in selected populations, whereas randomized trials have not definitively established benefit. Bleeding risk remains an important safety concern, and guideline panels have therefore not endorsed routine disease-modifying anticoagulation in sepsis (15–17). Despite increasing recognition of thrombo-inflammatory mechanisms in septic shock and widespread use of anticoagulation in critically ill patients, the net clinical effects of systemic heparin exposure remain uncertain (18, 19).

Using a large federated electronic health record network, this study evaluated the association between early systemic heparin exposure and mortality, thrombotic complications, organ dysfunction, and bleeding-related outcomes in patients with septic shock. It was hypothesized that early systemic heparin exposure would be associated with lower mortality and fewer thrombotic complications, while potentially increasing bleeding-related adverse events. This design focused on hard clinical endpoints captured in routine care rather than mechanistic biomarkers, allowing evaluation of both potential benefit and harm in a large contemporary septic shock population.

## 2 Materials and methods

### 2.1 Study design and ethical statement

We performed a retrospective cohort analysis using the TriNetX research network, a federated real-world data platform aggregating de-identified electronic health records (EHR) from academic and non-academic healthcare institutions. The study represents a secondary analysis of de-identified clinical data without intervention or patient contact. The Ethics Committee of the University of Lübeck confirmed that studies exclusively using TriNetX data are not subject to additional ethical approval requirements or informed consent. This assessment is in line with previous publications from the University of Lübeck and the University Hospital Schleswig-Holstein (20–22). The reporting of this study adheres to the STROBE guidelines for observational studies.

### 2.2 Study population and exposure definition

Adult patients (≥18 years) hospitalized with sepsis were identified using ICD-10 diagnosis codes and EHR-available Sepsis-3 surrogates: Septic shock was defined as sepsis (ICD-10 A41.x) combined with vasopressor therapy (norepinephrine, epinephrine, or vasopressin) and serum lactate ≥2 mmol/L. Accordingly, vasopressor exposure was a mandatory inclusion criterion, and no non-vasopressor comparator group was included in the present septic shock cohort. The sepsis episode index date corresponded to the infection diagnosis with a ±24-h window for organ dysfunction and vasopressor variables. Because complete SOFA components are not consistently available in federated EHR data, this definition represents an EHR-derived approximation of Sepsis-3 septic shock rather than a prospectively adjudicated diagnosis.

Patients receiving oral anticoagulants, including vitamin K antagonists or direct oral anticoagulants, were excluded to avoid comparator contamination. Early systemic heparin exposure was defined as unfractionated heparin or low-molecular-weight heparin within the first 48 h after the operational diagnosis of septic shock. Restricting exposure to this predefined window was intended to reduce immortal-time bias and ensure that heparin administration occurred during early septic shock management. Patients with heparin exposure during this window were compared with patients without documented heparin administration during the same period (Figure 1). Patients with heparin exposure during the predefined exposure window formed the exposed cohort; patients without heparin during that same window formed the reference (control) cohort. This time-anchored exposure definition was chosen to reduce the risk that later heparin-use merely reflected prolonged survival or delayed treatment escalation.

**Figure 1:**
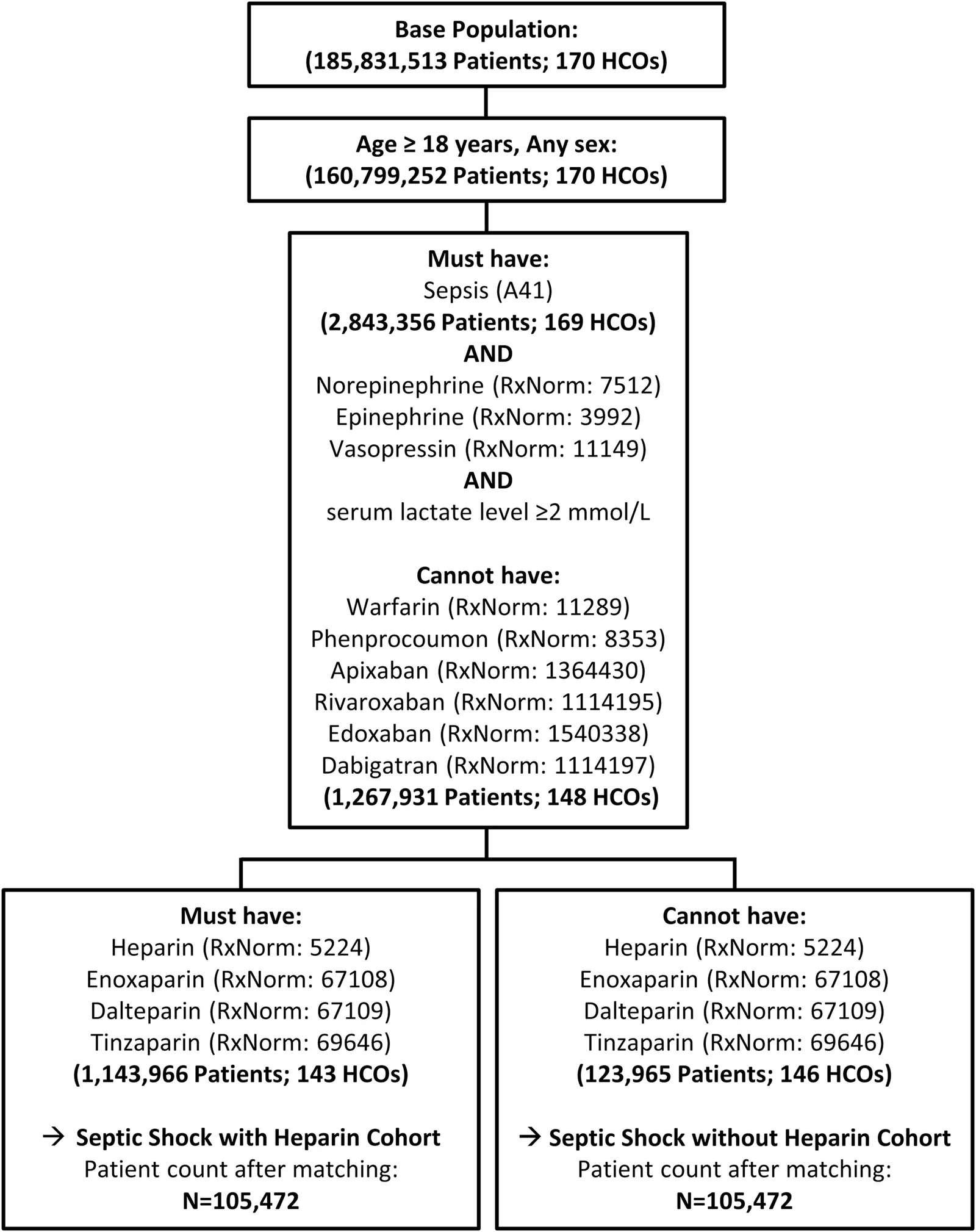
Flow diagram of cohort selection and propensity score matching. Flow chart of cohort selection from the TriNetX network. Septic shock was defined as sepsis (ICD-10 A41.x) in combination with vasopressor therapy and a serum lactate concentration ≥2 mmol/L. Patients receiving oral anticoagulants, including direct oral anticoagulants and phenprocoumon, were excluded. After 1:1 propensity score matching, two balanced cohorts were obtained: septic shock with early systemic heparin exposure (*n* = 105,472) and septic shock without heparin exposure (*n* = 105,472). HCO, healthcare organization.

No distinction could be made between prophylactic and therapeutic anticoagulation because dosing intensity, treatment duration, monitoring targets, and clinical indication are not consistently available within TriNetX. Accordingly, this analysis should be interpreted as evaluating early systemic heparin exposure rather than a predefined anticoagulation strategy. Heparin exposure may reflect thromboprophylaxis, therapeutic anticoagulation, extracorporeal circuit anticoagulation, suspected thrombotic complications, or other clinician-driven treatment decisions. Patient-level heparin dose, UFH monitoring parameters, longitudinal hemoglobin changes, and the extent to which renal or hepatic dysfunction influenced treatment decisions could not be reconstructed consistently within the federated dataset.

### 2.3 Cohort restrictions and outcome definitions

To reduce confounding by mandatory therapeutic anticoagulation, patients with extracorporeal membrane oxygenation or diagnoses suggesting acute thromboembolic disease requiring therapeutic anticoagulation (e.g., acute limb ischemia or pulmonary embolism) were excluded. Clinical outcomes were identified using predefined ICD-10 diagnosis codes and procedure codes recorded in the EHR (Table 1). Because laboratory values and clinical adjudication are not uniformly available across participating healthcare organizations, endpoints were defined as operational EHR phenotypes rather than adjudicated diagnoses according to contemporary guideline definitions. This is particularly relevant for DIC, thrombocytopenia, and major bleeding. DIC and thrombocytopenia were captured as separate, non-mutually exclusive EHR phenotypes using ICD-10 codes D65 and D69, respectively; therefore, individual patients could contribute to both outcomes.

**Table 1:** Definitions of clinical endpoints.

Outcomes		ICD-10 codes
Acute kidney injury	Acute Kidney Failure	N17
Acute liver injury	Hepatic failure, not elsewhere classified, Unspecified jaundice	K72, R17
Acute myocardial infarction (AMI)	Acute myocardial infarction; Subsequent ST elevation (STEMI) and non-ST elevation (NSTEMI) myocardial infarction	I21, I22
Arterial or venous thrombosis	Arterial embolism and thrombosis; Other venous embolism and thrombosis; Cerebral infarction; Acute myocardial infarction	I74, I82, I63, I21
Death	Mortality	Deceased
Disseminated intravascular Coagulation	Disseminated intravascular coagulation [defibrination syndrome]	D65
Exploratory endothelial-associated organ dysfunction composite (Endothelial organ dysfunction)	Purpura and other hemorrhagic conditions, Performance of Urinary Filtration (Single, Multiple, Intermittent <6, 6–18, >18 Hours Per Day), Deceased	D69, 5A1D00Z, 5A1D60Z, 5A1D70Z,5A1D80Z, 5A1D90Z, Deceased
Ischemic stroke	Cerebral infarction	I63
Major bleeding	Nontraumatic subarachnoid hemorrhage, Nontraumatic intracerebral hemorrhage, Other and unspecified, nontraumatic intracranial hemorrhage, Hematemesis, Melena, Gastrointestinal hemorrhage, unspecified Hemorrhage, not elsewhere classified	I60, I61, I62, K92.0, K92.1, K92.2, R58
Renal replacement therapy (RRT)	Performance of Urinary Filtration (Single, Multiple, Intermittent <6, 6–18, >18 Hours Per Day)	5A1D60Z, 5A1D00Z, 5A1D80Z, 5A1D70Z, 5A1D90Z
Thrombocytopenia	Purpura and other hemorrhagic conditions	D69
Thrombotic events	Other venous embolism and thrombosis, Arterial embolism and thrombosis	I82, I74
Transfusion requirement	Transfusion of Autologous/Non-Autologous Red Blood Cells into Peripheral/Central Vein or Central Artery	30233N1, 30233N0, 30243N1, 30243N0, 30263N1, 30263N0

The primary outcome was all-cause mortality within the predefined 30-day follow-up window. Secondary outcomes included acute kidney injury (AKI), renal replacement therapy (RRT), thrombocytopenia, transfusion requirement, DIC, major bleeding, acute liver injury, ischemic stroke, AMI, and additional arterial or venous thrombotic events. An exploratory endothelial-associated organ dysfunction composite (Endothelial Organ Dysfunction; EOD) was defined as the occurrence of death, RRT, or thrombocytopenia. This novel composite has not been externally validated and was considered a hypothesis-generating measure rather than a validated measure of endothelial injury.

Renal outcomes were assessed after the predefined 48-hour exposure window to reduce potential reverse causation; however, the exact temporal sequence between individual heparin administration and RRT initiation could not be reconstructed reliably. Endpoints were selected prior to data collection.

### 2.4 Propensity score matching and statistical analysis

Propensity score matching was applied to balance heparin-exposed and reference cohorts. Covariates were defined *a priori* based on clinical relevance and data availability and included age, sex, mechanical ventilation, serum lactate, infection source, major comorbidities, baseline AKI and RRT, thrombocytopenia, and other EHR-available severity markers measured before or independently of the exposure window. Comorbidities included chronic kidney disease, diabetes mellitus, liver disease, cardiovascular disease, heart failure, chronic ischemic heart disease, and malignancy. Infection foci included pneumonia, abdominal infection, and urinary tract infection. The choice of covariates was intended to capture both baseline comorbidity burden and EHR-available markers of septic shock severity in the absence of complete clinical severity scores. Baseline organ dysfunction variables, including AKI and thrombocytopenia, were considered only if documented at or before the index septic shock event to avoid adjustment for downstream consequences of heparin exposure. Clinician-driven treatment decisions and unmeasured confounders cannot be fully captured within observational EHR data and were therefore considered in the interpretation of results.

Nearest-neighbor 1:1 matching used a caliper width of 0.1 standard deviations of the logit of the propensity score. Covariate balance was assessed using standardized mean differences (SMD), with values <0.1 indicating adequate balance. Outcomes were evaluated in matched cohorts by calculating risk ratios, odds ratios, risk differences, and 95% confidence intervals. Where appropriate, time-to-event outcomes were analyzed using Kaplan–Meier estimates and Cox proportional hazards models. Risk ratios, odds ratios, risk differences, and hazard ratios were reported for heparin exposure versus no heparin exposure. Risk differences were calculated as the risk in the heparin cohort minus the risk in the non-heparin cohort. For Cox models, proportional hazards assumption statistics generated within the TriNetX platform were retained in the time-to-event table. Where the proportional hazards assumption was violated, hazard ratios were interpreted as summary estimates over the predefined follow-up period rather than as constant effects over time. Two-sided *p* < 0.05 was considered statistically significant. Analyses were performed within TriNetX. Figures were generated using GraphPad Prism 8.4.2. The graphical abstract was created using BioRender (Biorender.com; last accessed June 2026).

## 3 Results

### 3.1 Study cohorts and baseline characteristics

Before Propensity Score Matching, patients with septic shock receiving systemic heparin differed substantially from those without heparin exposure. Patients in the heparin cohort were slightly older and had higher prevalences of diabetes mellitus, chronic kidney disease, cardiovascular disease, liver disease, infection-related diagnoses, baseline AKI, thrombocytopenia, lactate differences, and invasive mechanical ventilation (Table 2). To address these differences, 1:1 Propensity Score Matching was performed, resulting in two balanced cohorts of 105,472 patients each. After matching, demographic characteristics, comorbidities, infection-related diagnoses, organ dysfunction markers, lactate levels, and severity-related variables were well balanced, with low SMDs across covariates (Table 2).

**Table 2:** Baseline characteristics and covariate balance before and after propensity score matching of septic shock patients with and without heparin therapy.

Variables	Before matching				After matching			
	Septic shock with heparin (*N* = 1,120,596) *N* (%)	Septic shock w/o heparin (*N* = 105,472) *N* (%)	*p*-value	SMD	Septic shock with heparin (*N* = 105,472) *N* (%)	Septic shock w/o heparin (*N* = 105,472) *N* (%)	*p*-value	SMD
Age at index [years; M(SD)]	62.4 (17.1)	61.7 (20.3)	<0.001	0.033	61.5 (20.2)	61.7 (20.3)	0.0058	0.011
Sex (Female)	496,410 (46.6%)	58,583 (47.9%)	<0.001	0.026	59,002 (48.2%)	58,582 (47.9%)	0.0893	0.007
Respiratory ventilation, <24 Consecutive hours	17,487 (1.64%)	672 (0.55%)	<0.001	0.105	490 (0.40%)	672 (0.55%)	0.051	0.022
Respiratory ventilation, 24–96 Consecutive hours	26,528 (2.49%)	883 (0.72%)	<0.001	0.141	686 (0.56%)	883 (0.72%)	0.0531	0.020
Respiratory ventilation, >96 Consecutive hours	21,291 (1.9%)	*738* (0.7%)	<0.001	0.128	738 (0.7%)	738 (0.7%)	0.922	<0.001
Renal replacement therapy (baseline)	265 (0.025%)	18 (0.015%)	0.0292	0.007	10 (0.008%)	18 (0.015%)	0.1305	0.006
Diabetes mellitus	352,294 (33.1%)	27,192 (22.2%)	<0.001	0.244	26,881 (22.0%)	27,192 (22.2%)	0.1297	0.006
Urinary tract infection	223,525 (21.0%)	21,069 (17.2%)	<0.001	0.096	20,915 (17.1%)	21,065 (17.2%)	0.4212	0.003
Chronic kidney disease	246,354 (23.1%)	17,761 (14.5%)	<0.001	0.222	17,509 (14.3%)	17,757 (14.5%)	0.1534	0.006
Pneumonia	186,279 (17.5%)	15,822 (12.9%)	<0.001	0.127	15,574 (12.7%)	15,818 (12.9%)	0.1402	0.006
Liver disease	178,651 (16.8%)	15,566 (12.7%)	<0.001	0.114	14,979 (12.2%)	15,562 (12.7%)	0.0004	0.014
Chronic ischemic heart disease	270,447 (25.4%)	14,441 (11.8%)	<0.001	0.354	14,640 (12.0%)	14,441 (11.8%)	0.2138	0.005
Heart failure	225,228 (21.1%)	12,071 (9.9%)	<0.001	0.315	12,141 (9.9%)	12,071 (9.9%)	0.6356	0.002
Acute kidney injury (baseline)	250,260 (23.5%)	11,573 (9.46%)	<0.001	0.385	11,255 (9.20%)	11,573 (9.46%)	0.0271	0.009
Diverticular disease	126,952 (11.9%)	6,448 (5.27%)	<0.001	0.239	6,491 (5.31%)	6,448 (5.27%)	0.6977	0.002
Thrombocytopenia (baseline)	110,024 (10.3%)	6,696 (5.47%)	<0.001	0.181	6,428 (5.25%)	6,696 (5.47%)	0.0162	0.010
Peritonitis	24,283 (2.28%)	1,765 (1.44%)	<0.001	0.062	1,330 (1.09%)	1,765 (1.44%)	0.061	0.032
Baseline serum lactate [M (SD)]	3.77 (1.36)	3.00 (2.09)	<0.001	0.244	3.12 (1.71)	3.00 (2.09)	0.073	0.019

w/o, without; SMD, standardized mean difference (values <0.1 indicate adequate covariate balance).

### 3.2 Risk-based analyses of clinical outcomes

Heparin exposure was associated with a significantly lower risk of 30-day mortality compared with no heparin exposure (*p* < 0.001), corresponding to an absolute risk reduction of 5.5% (Table 3; Figure 2A). AKI occurred more frequently among patients receiving heparin (*p* < 0.001), and RRT was likewise higher in the heparin cohort (*p* < 0.001). Thrombocytopenia was also more common with heparin exposure (*p* < 0.001; Table 3). In contrast, DIC occurred less frequently in the heparin cohort (*p* < 0.001). The exploratory endothelial-associated organ dysfunction composite did not differ significantly between cohorts (*p* = 0.064). Regarding safety outcomes, major bleeding was slightly more frequent with heparin (*p* = 0.002), transfusion requirement was substantially higher (*p* < 0.001), and acute liver injury was similar between groups (*p* = 0.683; Table 3).

**Table 3:** Risk-based analyses of clinical outcomes.

Outcome	Septic shock with heparin (*N* = 105,472)		Septic shock w/o heparin (*N* = 105,472)										
	N of outcomes	Risk (%)	N of outcomes	Risk (%)	Risk Ratio	RR 95% CI	Odds Ratio	OR 95% CI	Risk Difference	RD 95% CI	z	*p*-value	Cohen’s D
Acute kidney injury	24,465	23.2	14,753	14.0	1.658	1.628–1.689	1.857	1.816–1.899	0.092	0.089–0.095	54.354	**<0** **.** **001**	0.28
Acute liver injury	4,300	4.1	4,263	4.0	1.009	0.968–1.051	1.009	0.966–1.054	0.000	−0.001–0.002	0.408	0.683	0.005
Acute myocardial infarction (AMI)	2,170	2.06	6,228	5.90	0.348	0.332–0.366	0.337	0.321–0.354	−0.038	−0.040 to −0.037	−45.411	**<0** **.** **001**	−0.58
Arterial or venous thrombosis	6,291	5.97	16,171	15.33	0.389	0.378–0.400	0.357	0.346–0.368	−0.094	−0.096 to −0.091	−70.558	**<0** **.** **001**	−0.52
Death	16,103	15.3	21,901	20.8	0.735	0.722–0.749	0.688	0.672–0.703	−0.055	−0.058 to −0.052	−32.847	**<0** **.** **001**	−0.17
Disseminated intravascular coagulation	789	0.7	1,001	0.9	0.788	0.718–0.865	0.787	0.716–0.864	−0.002	−0.003 to −0.001	−5.032	**<0** **.** **001**	−0.13
Endothelial Organ Dysfunction	26,239	24.9	26,607	25.2	0.986	0.972–1.001	0.982	0.962–1.001	−0.003	−0.007–0.000	−1.849	0.064	−0.01
Ischemic stroke	2,696	2.56	4,249	4.03	0.635	0.605–0.665	0.627	0.597–0.658	−0.015	−0.016 to −0.013	−18.966	**<0** **.** **001**	−0.25
Major bleeding	6,675	6.3	6,331	6.0	1.054	1.020–1.090	1.058	1.021–1.096	0.003	0.001–0.005	3.114	**0** **.** **002**	0.03
Renal replacement therapy (RRT)	5,556	5.3	2,874	2.7	1.933	1.850–2.021	1.985	1.896–2.078	0.025	0.024–0.027	29.813	**<0** **.** **001**	0.36
Thrombocytopenia	9,645	9.1	6,238	5.9	1.546	1.499–1.594	1.601	1.549–1.655	0.032	0.030–0.035	28.113	**<0** **.** **001**	0.24
Thrombotic events	1,883	1.79	7,792	7.39	0.242	0.230–0.254	0.230	0.219–0.242	−0.056	−0.058 to −0.054	−62.060	**<0** **.** **001**	−0.78
Transfusion requirement	9,017	8.5	3,558	3.4	2.534	2.440–2.632	2.678	2.573–2.786	0.052	0.050–0.054	50.200	**<0** **.** **001**	0.51

w/o, without; RR, risk ratio; OR, odds ratio; RD, risk difference; CI, confidence interval. Risk ratios, odds ratios, and risk differences are reported for heparin exposure versus no heparin exposure. Risk differences were calculated as risk in the heparin cohort minus risk in the non-heparin cohort. Values <1 for RR and OR indicate lower risk in the heparin cohort. Endothelial organ dysfunction refers to the exploratory endothelial-associated organ dysfunction composite.

*Bold values indicate significance.

**Figure 2:**
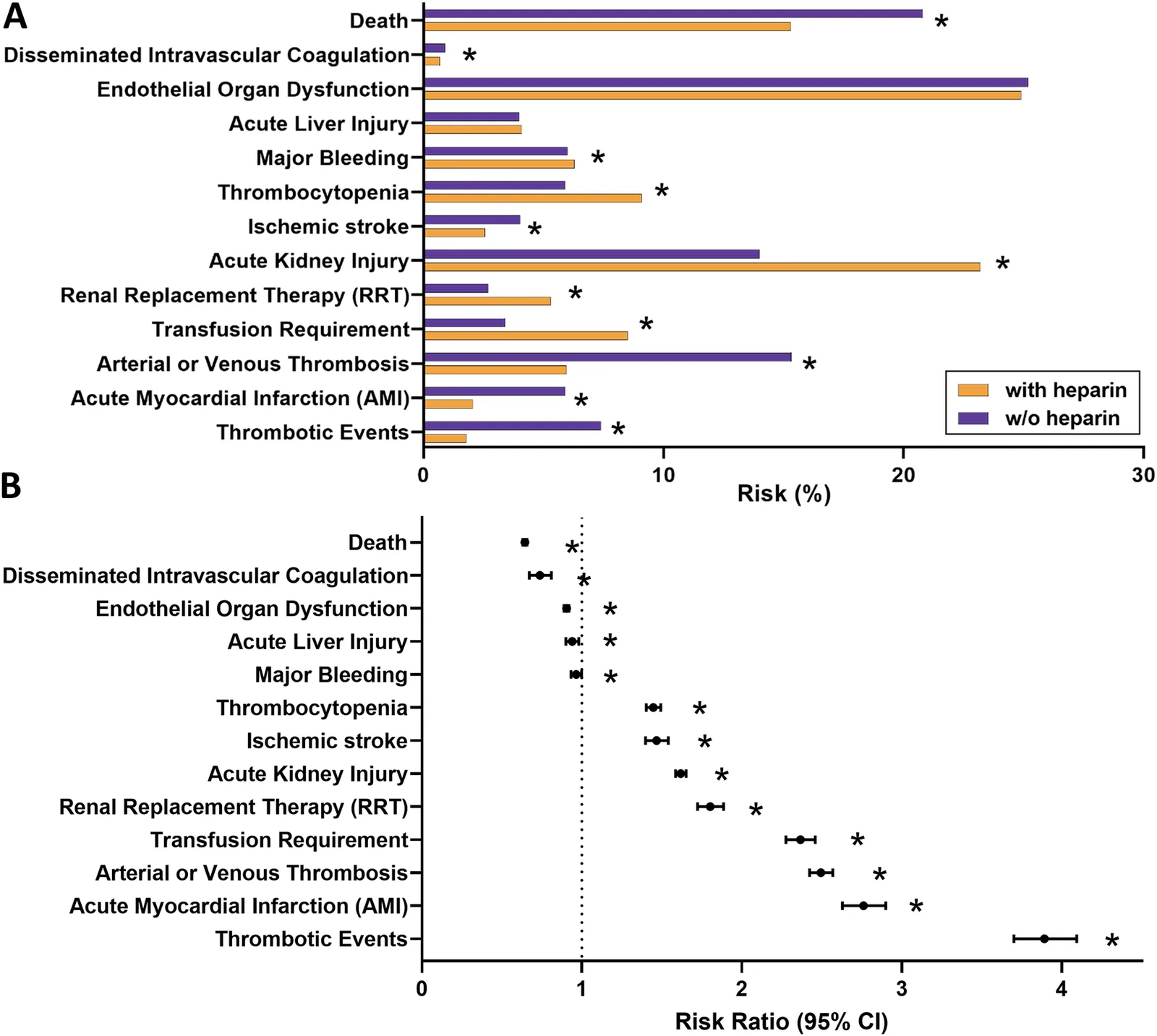
Risk-based comparison of clinical outcomes in propensity score-matched cohorts. **(A)** Absolute risk of clinical outcomes in patients with septic shock receiving systemic heparin compared with matched patients without heparin exposure. **(B)** Forest plot of risk ratios (RR) with 95% confidence intervals for all outcomes, comparing heparin exposure versus no heparin. Systemic heparin exposure was associated with lower risks of mortality, disseminated intravascular coagulation, and thrombotic complications, but higher risks of renal outcomes, thrombocytopenia, and transfusion requirement. No significant differences were observed for acute liver injury or the exploratory endothelial-associated organ dysfunction composite. Analyses were performed after 1:1 propensity score matching (*n*  =  105,472 per cohort). Detailed estimates are provided in Table 3.

Thrombotic complications occurred consistently less frequently among heparin-exposed patients (Figure 2A). Ischemic stroke and AMI were both reduced (both *p* < 0.001), as were overall thrombotic events and the composite endpoint of arterial or venous thrombosis (both *p* < 0.001; Table 3). Risk ratio analyses mirrored the absolute risk findings (Figure 2B). Heparin exposure was associated with lower relative risks of death, DIC, and thrombotic outcomes; no significant difference in acute liver injury or the exploratory composite; and higher relative risks of AKI, RRT, thrombocytopenia, major bleeding, and transfusion requirement. The largest relative effect estimates were observed for thrombotic outcomes (RR range 0.24–0.64; all *p* < 0.001).

### 3.3 Time-to-event analyses

Kaplan–Meier analyses were consistent with risk-based results (Table 4; Figures 3, 4). Heparin exposure was associated with reduced hazards of death (Figure 3A), DIC (*p* < 0.001), and the exploratory endotheliale composite (Figure 3B). Reduced hazards were also observed for ischemic stroke (Figure 3C), overall thrombotic events (Figure 3D), AMI (Figure 3E), and arterial or venous thrombosis (Figure 3F; Table 4). Renal and hematologic complications were more frequent among patients receiving heparin. Heparin exposure was associated with increased hazards of thrombocytopenia (Figure 4A), AKI (Figure 4B), RRT (Figure 4C), and transfusion requirement (Figure 4D; Table 4).

**Table 4:** Time-to-event analyses.

Outcome	Septic shock with heparin (*N* = 105,472)		Septic shock without heparin (*N* = 105,472)		Log-rank test		Cox proportional hazards model		Proportional hazards assumption test	
	N of outcomes	Survival (%)	N of outcomes	Survival (%)	*χ*²	*p*-value	Hazard Ratio (HR)	95% CI	χ²	*p*-value
Acute kidney injury	24,465	75.80	14,753	84.62	2231.389	<0.001	1.618	1.585–1.651	103.880	<0.001
Acute liver injury	4,300	95.71	4,263	95.51	8.321	0.004	0.939	0.900–0.980	32.187	<0.001
Acute myocardial infarction (AMI)	2,170	97.94	6,228	94.10	1812.619	<0.001	0.362	0.345–0.380	0.316	0.574
Arterial or venous thrombosis	6,291	94.03	16,171	84.67	4077.383	<0.001	0.401	0.389–0.413	48.981	<0.001
Death	16,103	84.05	21,901	77.11	1827.468	<0.001	0.644	0.631–0.657	820.556	<0.001
Disseminated intravascular coagulation	789	99.21	1,001	98.96	41.488	<0.001	0.737	0.671–0.809	26.061	<0.001
Endothelial organ dysfunction	26,239	74.25	26,607	72.49	133.686	<0.001	0.904	0.889–0.919	259.650	<0.001
Ischemic stroke	2,696	97.44	4,249	95.97	245.535	<0.001	0.682	0.649–0.715	110.480	<0.001
Major bleeding	6,675	93.22	6,331	93.17	3.934	0.047	0.965	0.933–0.999	112.925	<0.001
Renal replacement therapy (RRT)	5,556	94.45	2,874	96.92	678.694	<0.001	1.803	1.723–1.886	8.609	0.003
Thrombocytopenia	9,645	90.33	6,238	93.34	527.292	<0.001	1.447	1.402–1.494	44.044	<0.001
Thrombotic events	1,883	98.21	7,792	92.61	3263.530	<0.001	0.257	0.244–0.270	0.511	0.475
Transfusion requirement	9,017	90.89	3,558	96.24	2019.332	<0.001	2.366	2.276–2.459	261.588	<0.001

w/o, without; CI, confidence interval. Survival probabilities refer to the end of the predefined follow-up period. Log-rank χ² statistics and *p*-values compare Kaplan–Meier curves between cohorts. Hazard ratios (HR) were estimated using Cox proportional hazards models and are reported for heparin exposure versus no heparin exposure. HR values <1 indicate lower hazard in the heparin cohort. The final two columns report the test statistics and *p*-values for assessment of the proportional hazards assumption generated within the TriNetX platform. Endothelial organ dysfunction refers to the exploratory endothelial-associated organ dysfunction composite.

**Figure 3:**
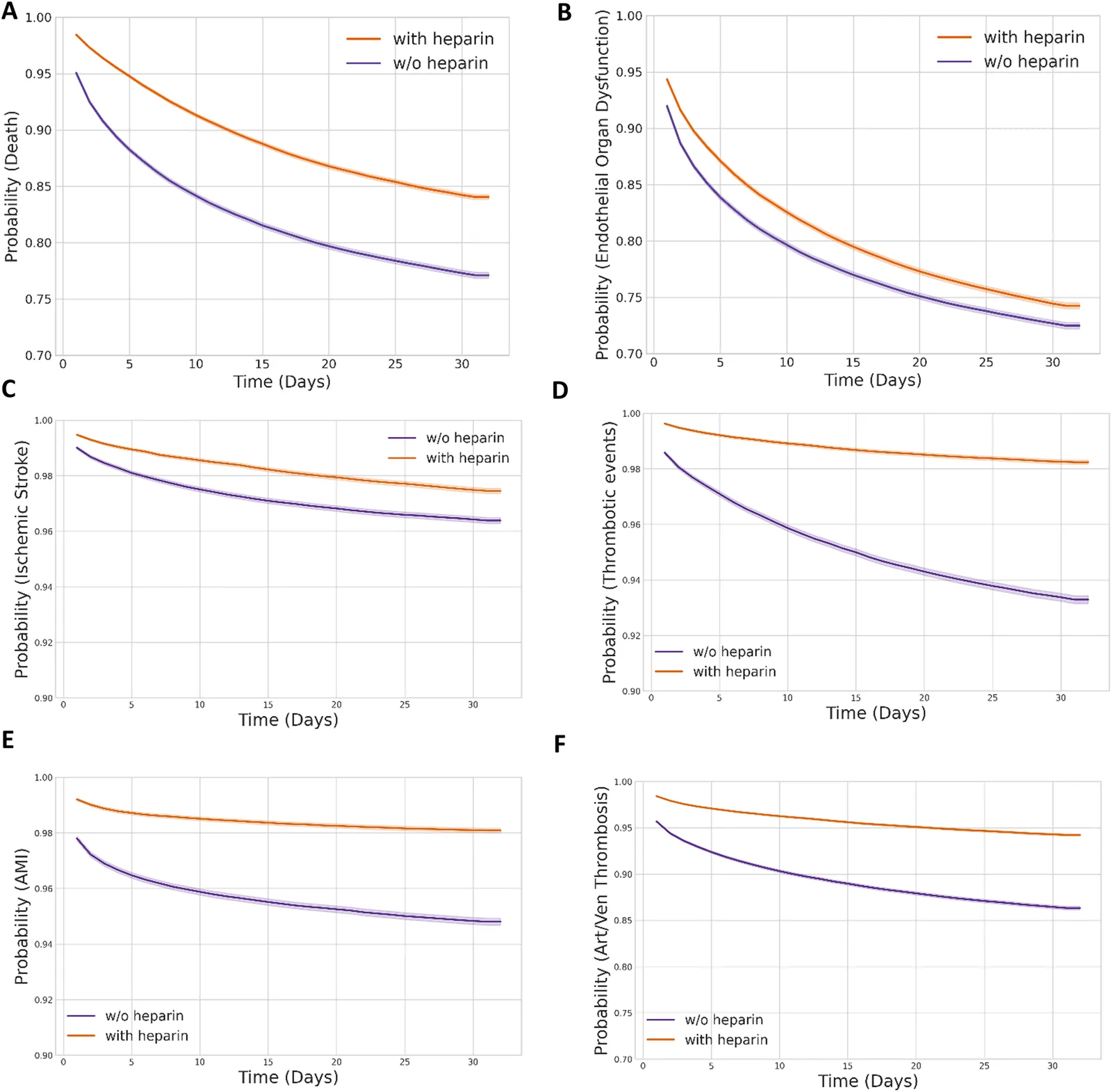
Kaplan–Meier analyses of mortality and thrombotic outcomes. Kaplan–Meier curves for time-to-event outcomes in propensity score-matched patients with septic shock receiving systemic heparin versus no heparin exposure, including **(A)** overall 30-day survival, **(B)** endothelial organ dysfunction (exploratory endothelial-associated organ dysfunction composite), **(C)** ischemic stroke, **(D)** overall thrombotic events, **(E)** acute myocardial infarction, and **(F)** arterial or venous thrombosis. Hazard ratios were estimated using Cox proportional hazards models and statistical significance was assessed using log-rank tests (Table 4). Systemic heparin exposure was associated with lower mortality and lower rates of thrombotic complications across all thrombotic endpoints.

**Figure 4:**
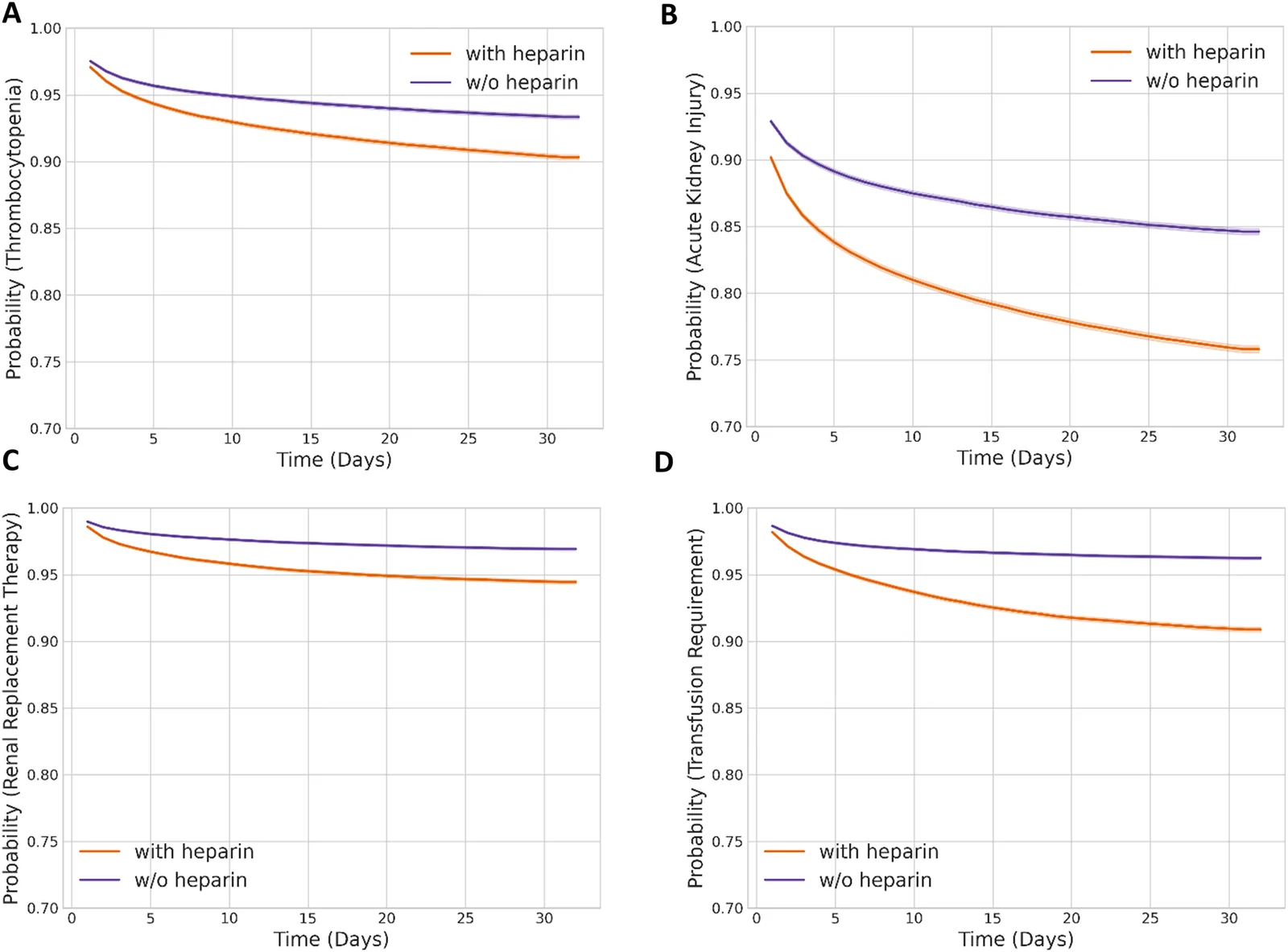
Kaplan–Meier analyses demonstrating increased renal and hematologic complications with systemic heparin**.** Kaplan–Meier curves for time-to-event outcomes in propensity score-matched patients with septic shock receiving systemic heparin versus no heparin exposure, including **(A)** thrombocytopenia, **(B)** acute kidney injury, **(C)** renal replacement therapy, and **(D)** transfusion requirement. Hazard ratios were estimated using Cox proportional hazards models and statistical significance was assessed using log-rank tests (Table 4). Systemic heparin exposure was associated with increased hazards of thrombocytopenia, acute kidney injury, renal replacement therapy, and transfusion requirement.

Overall, time-to-event analyses confirmed the pattern of lower mortality, DIC, and thrombotic complications, accompanied by higher renal dysfunction, thrombocytopenia, and transfusion requirement. Across risk-based and time-to-event analyses, the direction of associations was consistent for the main outcome domains. Lower mortality, DIC, and thrombotic complications were observed alongside higher renal and hematologic complications and transfusion requirement, supporting the interpretation of a clinically relevant “benefit vs. harm” profile rather than a uniform treatment-associated advantage.

## 4 Discussion

In this large propensity-matched real-world analysis of patients with septic shock, systemic heparin exposure was associated with lower 30-day mortality and lower DIC incidence, but higher rates of renal dysfunction, thrombocytopenia, bleeding, and transfusion. These findings delineate a clinically relevant trade-off between lower mortality and thrombotic complications on the one hand and increased renal and bleeding-related complications on the other. The principal associations and the resulting clinical trade-off are summarized in Figure 5. They also address uncertainty regarding anticoagulation in septic shock, a condition characterized by thrombo-inflammatory dysregulation, immunothrombosis, microvascular dysfunction, and DIC. Although the observed mortality reduction was substantial, the magnitude of this association should be interpreted cautiously given the observational study design and the possibility of residual confounding despite extensive propensity matching.

**Figure 5:**
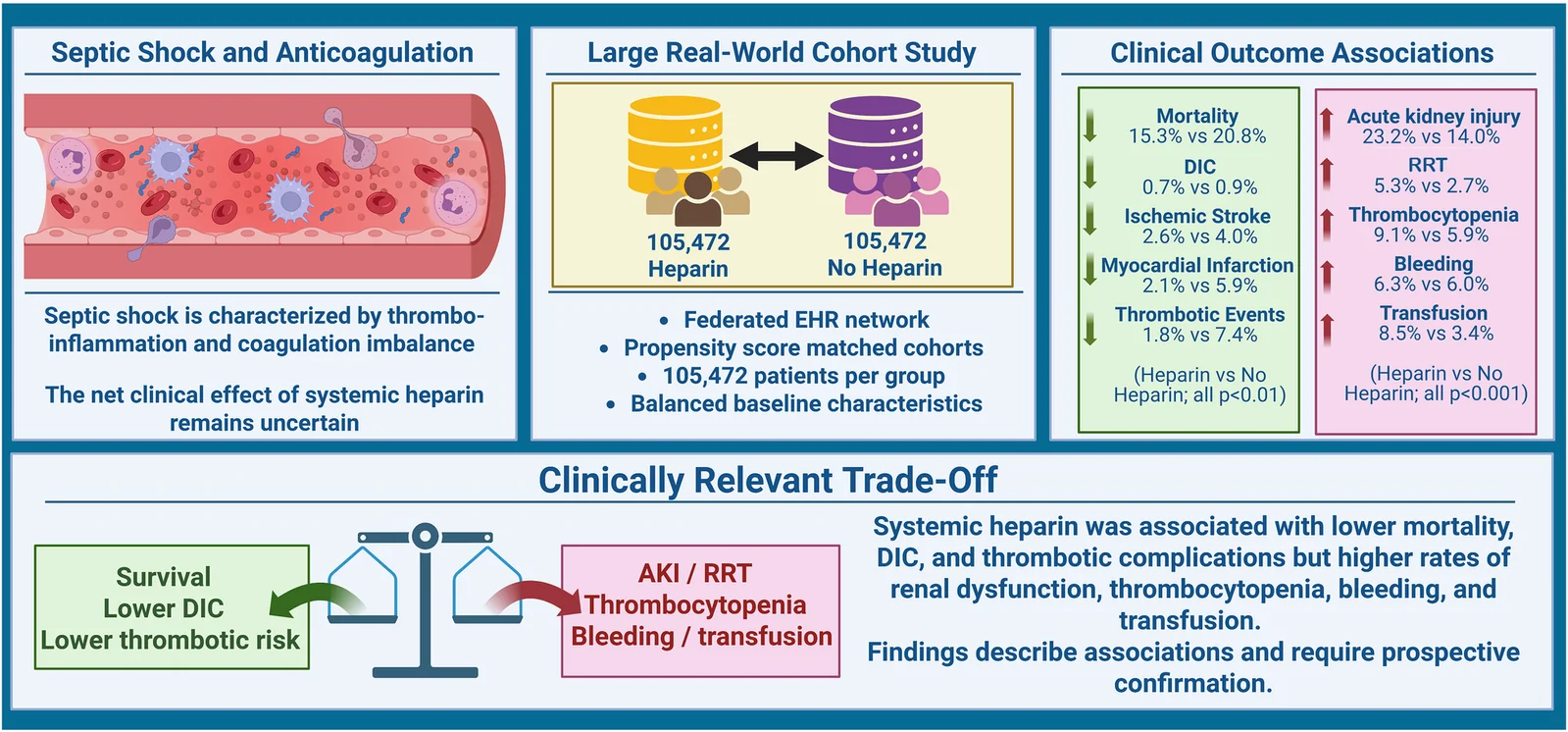
Graphical summary of the principal associations between early systemic heparin exposure and clinical outcomes in septic shock. The study compared propensity score-matched cohorts of 105,472 patients with septic shock receiving early systemic heparin exposure and 105,472 patients without heparin exposure. Systemic heparin exposure was associated with lower mortality, disseminated intravascular coagulation (DIC), ischemic stroke, myocardial infarction, and thrombotic events, but higher rates of acute kidney injury (AKI), renal replacement therapy (RRT), thrombocytopenia, bleeding, and transfusion. The findings illustrate a clinically relevant trade-off between favorable and unfavorable outcome associations and require prospective confirmation. (EHR: Electronic Health Record).

Our analysis also demonstrated lower rates of ischemic stroke, AMI, and broader arterial or venous thrombotic events among heparin-exposed patients. This is consistent with the systemic prothrombotic state of sepsis, driven by inflammation, endothelial injury, and dysregulated coagulation, and with population data showing increased risks of myocardial infarction and ischemic stroke during and after sepsis (11, 12, 23).

The pattern observed - lower mortality and lower incidence of DIC with systemic heparin but higher rates of AKI, dialysis, thrombocytopenia, bleeding and transfusion—seems consistent with a mechanistically complex interaction with thrombo-inflammatory endothelial pathways rather than a unidirectional benefit or harm.

Experimental and translational studies suggest that heparin and related glycosaminoglycans may inhibit heparanase, reduce glycocalyx shedding, blunt leukocyte-endothelial interactions, and interfere with complement activation and platelet aggregation, thereby attenuating microvascular thrombosis and consumptive coagulopathy in systemic inflammation (13, 14, 24, 25). Multiple thrombo-inflammatory pathways contribute to endothelial dysfunction, microvascular thrombosis, and DIC in septic shock (7, 9, 10, 26, 27).

Clinical evidence on heparin in sepsis has been heterogeneous (19). Older randomized and observational studies were underpowered or inconsistent, and meta-analyses have reported mixed findings, ranging from signals toward reduced mortality or DIC to no definitive survival advantage (13, 16, 19, 28, 29). More recent studies and meta-analyses suggest potential benefit in selected patients with sepsis-associated coagulopathy while emphasizing bleeding risk and heterogeneity (30–33). Contemporary reviews similarly argue that anticoagulant therapy may be most relevant in biologically enriched subgroups with sepsis-associated coagulopathy or DIC, whereas routine use in unselected septic populations remains unsupported (34).

Our findings mirror this complex clinical picture: lower mortality and DIC were accompanied by increased bleeding and transfusion, consistent with known anticoagulant trade-offs (30, 32, 33). The apparent paradox of lower mortality despite higher rates of AKI, RRT, thrombocytopenia, and transfusion suggests that survival and non-fatal organ dysfunction may not necessarily move in parallel in observational septic shock cohorts and warrants careful interpretation.

In routine intensive care practice, absence of pharmacologic anticoagulation may reflect active bleeding, severe thrombocytopenia, coagulopathy, recent surgery, procedural risk, or clinician judgment rather than clinical stability. Before matching, the heparin cohort had a substantially greater burden of comorbidity and illness severity, indicating preferential use in clinically complex patients. Although propensity matching improved measured balance, residual confounding by indication cannot be excluded.

The present findings may therefore inform risk-stratified investigations of heparin in patients with early coagulopathy or elevated thrombotic risk, but should not be interpreted as treatment recommendations. These observations may inform future trials by identifying enriched subgroups, relevant efficacy and safety endpoints, and timing strategies to reduce confounding by indication (13, 16, 19, 28, 35).

Importantly, all associations are non-causal and descriptive of real-world treatment patterns. Heparin has biological effects beyond anticoagulation, including modulation of thrombo-inflammatory pathways, endothelial protection, and attenuation of consumptive coagulopathy (13, 36). At the same time, bleeding and transfusion are expected adverse effects of systemic anticoagulation and have been reported in sepsis studies (19). Survivor and competing-risk bias may contribute to higher rates of non-fatal complications, because the higher survival observed in the heparin-exposed cohort results in a longer period at risk for outcomes such as AKI, thrombocytopenia, RRT, and transfusion (37, 38). Consequently, part of the observed excess in these secondary outcomes may reflect differential survival opportunity rather than a direct adverse effect of heparin. A formal competing-risk analysis treating death as a competing event could not be performed within the available TriNetX analysis framework. Therefore, the cumulative incidence of non-fatal outcomes should be interpreted cautiously, as differential mortality between cohorts may have influenced their observed frequency. Confounding by indication may also favor heparin use in patients with early coagulopathy or high thrombotic risk, a subgroup in which benefit may be more pronounced despite increased adverse events (33). Finally, the higher rates of AKI and RRT in the heparin-exposed cohort should not be interpreted as evidence of direct heparin-associated nephrotoxicity. In critically ill patients, initiation of continuous RRT may itself lead to heparin exposure for extracorporeal circuit anticoagulation; therefore, RRT may precede and contribute to heparin administration rather than represent a consequence of it. The temporal sequence between RRT initiation and individual heparin administration could not be reconstructed with sufficient granularity to perform a reliable sensitivity analysis excluding early RRT initiation. Accordingly, the observed associations with renal outcomes may be substantially influenced by reverse causality and CRRT-associated anticoagulation practices rather than a direct adverse renal effect of heparin.

Several limitations should be considered. One major limitation is the inability to distinguish prophylactic from therapeutic anticoagulation. Dosing intensity, treatment indication, monitoring targets, and treatment duration are not consistently available within TriNetX; therefore, this study evaluates early systemic heparin exposure rather than a predefined anticoagulation strategy.

Because prophylactic and therapeutic regimens differ in treatment indication, anticoagulant intensity, and safety profile, pooling these exposures limits attribution of the observed mortality, bleeding, and transfusion associations to any specific dosing strategy. Accordingly, the present findings cannot define the relative benefit-risk profile or clinical utility of prophylactic versus therapeutic heparin regimens.

Heparin administration may reflect thromboprophylaxis, therapeutic anticoagulation, extracorporeal circuit anticoagulation, suspected thrombosis, or other clinician-driven decisions. Furthermore, the severity of anemia around bleeding events, patient-level heparin dosing, and UFH monitoring parameters could not be assessed consistently. Although chronic kidney disease and liver disease were included in propensity matching, the extent to which renal or hepatic dysfunction influenced heparin initiation, dose selection, monitoring, or treatment duration cannot be determined. These limitations restrict causal interpretation of the observed bleeding and transfusion associations.

Residual confounding, including confounding by indication and survivor bias, cannot be fully excluded despite propensity matching. Although chronic kidney disease and heart failure were included in the matching model, other chronic inflammatory conditions such as COPD, rheumatoid disease, and vasculitis were not separately accounted for and may therefore contribute to residual confounding. In addition, interindividual variability in heparin response related to inflammatory activity and antithrombin availability could not be assessed, as CRP and antithrombin levels were not incorporated consistently into the present analysis. Furthermore, although baseline thrombocytopenia was included in the matching model, laboratory-based coagulation parameters such as platelet count, PT-INR, aPTT, and D-dimer were not available with sufficient consistency across participating healthcare organizations. Therefore, residual differences in baseline coagulopathy between cohorts cannot be excluded. Direct mechanistic biomarkers of endothelial injury, thrombo-inflammatory activation, and microvascular dysfunction are not routinely captured in federated EHR datasets. These constraints underscore the complementary role of large-scale real-world evidence, which can evaluate clinically relevant endpoints across heterogeneous populations but requires careful design and confounding control (37, 39–42). Accordingly, this study relies on clinically accessible surrogate outcomes that are biologically plausible but imperfect proxies for endothelial injury and thrombo-inflammatory activity (14, 25, 30).

Several outcomes were defined using administrative codes rather than laboratory-based clinical criteria. Septic shock itself was operationally defined using EHR-based surrogate criteria for Sepsis-3 diagnoses, thus, phenotype misclassification cannot be excluded. This is particularly relevant for DIC, thrombocytopenia, and major bleeding, for which coding practices may vary between institutions. Moreover, overlap between the DIC and thrombocytopenia-related phenotypes cannot be excluded, and the D69 category represents a broader coding-based phenotype rather than laboratory-confirmed thrombocytopenia according to a predefined platelet-count threshold. The exploratory endothelial-associated organ dysfunction composite has not been externally validated and should therefore be interpreted as hypothesis-generating. SOFA scores were unavailable, although illness severity was indirectly addressed using treatment-based and diagnostic proxies. In addition, proportional hazards assumptions were violated for several time-to-event outcomes. Accordingly, the corresponding Cox hazard ratios should be interpreted as overall summary estimates across the 30-day follow-up rather than as evidence of a constant relative hazard throughout the observation period. Finally, large sample size increases precision but may render small absolute differences statistically significant, while clinically relevant subgroup effects may be diluted in aggregated analyses.

Prospective randomized trials and complementary translational studies combining biomarker measurement with pragmatic trial designs are needed to clarify treatment timing, patient selection, and mechanisms (7, 43, 44). As with all observational analyses, these associations require confirmation in adequately powered randomized or biomarker-informed interventional studies. Against this background, the present data provide pragmatic evidence of lower mortality, DIC, and thrombotic complications with early systemic heparin exposure, accompanied by increased renal dysfunction, bleeding, and transfusion, underscoring the need for carefully targeted prospective evaluation.

## 5 Conclusion

In this large propensity-matched real-world cohort of patients with septic shock, early systemic heparin exposure was associated with lower 30-day mortality, reduced rates of disseminated intravascular coagulation and thrombotic complications, but increased rates of renal dysfunction, thrombocytopenia, bleeding, and transfusion. These findings highlight a clinically relevant trade-off between potential survival benefit and treatment-related adverse events in a population characterized by profound thrombo-inflammatory dysregulation, immunothrombosis, and microvascular dysfunction. While causal inference is not possible in this observational analysis, the observed associations are biologically consistent with a potential role of heparin in modulating coagulation-dependent thrombo-inflammatory processes in septic shock. Prospective randomized studies are needed to define optimal patient selection, treatment timing, and the balance between potential benefit and harm (35).

## Data Availability

The original contributions presented in the study are included in the article/Supplementary Material, further inquiries can be directed to the corresponding author.
